# Availability, procurement, training, usage, maintenance and complications of electrosurgical units and laparoscopic equipment in 12 African countries

**DOI:** 10.1002/bjs5.50255

**Published:** 2020-01-27

**Authors:** R. M. Oosting, L. S. G. L. Wauben, J. K. Madete, R. S. Groen, J. Dankelman

**Affiliations:** ^1^ Department of Biomechanical Engineering Delft University of Technology Delft the Netherlands; ^2^ Innovations in Care Research Centre Rotterdam University of Applied Sciences Rotterdam the Netherlands; ^3^ Department of Electrical and Electronic Engineering School of Engineering and Technology, Kenyatta University Nairobi Kenya; ^4^ Department of Gynecology and Obstetrics Johns Hopkins University Baltimore Maryland USA

## Abstract

**Background:**

Strategies are needed to increase the availability of surgical equipment in low‐ and middle‐income countries (LMICs). This study was undertaken to explore the current availability, procurement, training, usage, maintenance and complications encountered during use of electrosurgical units (ESUs) and laparoscopic equipment.

**Methods:**

A survey was conducted among surgeons attending the annual meeting of the College of Surgeons of East, Central and Southern Africa (COSECSA) in December 2017 and the annual meeting of the Surgical Society of Kenya (SSK) in March 2018. Biomedical equipment technicians (BMETs) were surveyed and maintenance records collected in Kenya between February and March 2018.

**Results:**

Among 80 participants, there were 59 surgeons from 12 African countries and 21 BMETs from Kenya. Thirty‐six maintenance records were collected. ESUs were available for all COSECSA and SSK surgeons, but only 49 per cent (29 of 59) had access to working laparoscopic equipment. Reuse of disposable ESU accessories and difficulties obtaining carbon dioxide were identified. More than three‐quarters of surgeons (79 per cent) indicated that maintenance of ESUs was available, but only 59 per cent (16 of 27) confirmed maintenance of laparoscopic equipment at their centre.

**Conclusion:**

Despite the availability of surgical equipment, significant gaps in access to maintenance were apparent in these LMICs, limiting implementation of open and laparoscopic surgery.

## Graphical abstract

This study investigated the current availability, procurement, training, usage, maintenance and complications in low‐ and middle‐income countries (LMICs) of two types of surgical equipment: the electrosurgical unit and laparoscopic equipment. Despite the availability of surgical equipment, surgeons still encountered complications during use of both types of surgical equipment. Reducing gaps in the availability of training and access to maintenance, together with the implementation of equipment that fits current use in LMICs, are important steps in reaching global implementation of open and laparoscopic surgery.

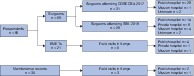

Adoption of new technology is complex

## Abstract (es)

**Antecedentes:**

En países de bajos y medianos ingresos (*low‐ and middle‐income countries*, LMIC) hay que desarrollar estrategias de futuro para incrementar la disponibilidad de equipos quirúrgicos, adquisición, capacitación, uso, mantenimiento y complicaciones relacionadas con las unidades electroquirúrgicas (*electrosurgical unit*, ESU) y los equipos de laparoscopia.

**Métodos:**

Se realizó una encuesta entre los cirujanos que asistieron a la reunión anual del Colegio de Cirujanos de África Oriental, Central y Meridional (*College Of Surgeons of East, Central and Southern Africa*, COSECSA) en diciembre de 2017 y a la reunión anual de la Sociedad Quirúrgica de Kenia (*Surgical Society of Kenya*, SSK) en marzo de 2018. Se encuestaron también a los técnicos de equipos biomédicos (*Biomedical Equipment Technicians*, BMET) y se recopilaron los registros de mantenimiento en Kenia entre febrero y marzo de 2018.

**Resultados:**

Participaron 80 sujetos, 59 cirujanos de 11 países africanos y 21 BMET de Kenia. Se recopilaron 36 registros de mantenimiento. Todos los cirujanos de COSECSA y SSK disponían de ESU, pero menos de la mitad (49%) disponían de equipos de laparoscopia. Como principales problemas se detectaron la reutilización de accesorios desechables en las ESU y las dificultades para disponer de CO2. Más de las tres cuartas partes (78%) de los cirujanos contaban con equipos de mantenimiento para las ESU, pero solo el 59% disponía de mantenimiento para los equipos de laparoscopia en su centro.

**Conclusión:**

A pesar de la disponibilidad de equipos quirúrgicos, en estos LMIC se detectaron serias dificultades en su mantenimiento, hecho que limita la implementación de la cirugía abierta y laparoscópica.

## Introduction

There is an increased need for surgery in low‐ and middle‐income countries (LMICs). An estimated five billion people in LMICs do not have access to safe and affordable surgery^1^, ^2^. In high‐income countries, laparoscopic surgery is used widely and, compared with open surgery, is associated with decreased risks of infection and blood loss, and more rapid return to work^3^. In LMICs, diagnostic laparoscopy could replace expensive modern diagnostic modalities, such as MRI and CT^4^. There are, however, major clinical, economic and infrastructural barriers to widespread implementation of laparoscopic surgery in LMICs, including limited access to trained laparoscopic surgeons, the high cost of laparoscopic equipment, need for general anaesthesia, and limited resources to handle maintenance issues^3^. Despite these barriers, successful implementation of laparoscopic surgery has shown significantly improved outcomes in LMICs^5^.

Adequate surgical equipment is vital for the provision of safe surgical care. Shortages of surgical equipment have been found in previous studies^6^, ^7^, ^8^, ^9^, ^10^, ^11^, ^12^, ^13^ from Nigeria, Cameroon, Sierra Leone, Somalia, Ethiopia and Malawi. To ensure that surgical equipment is available, a system supporting the equipment needs to be in place so that the right equipment is procured, used and maintained as intended, following appropriate training along with a secure supply chain of consumables^14^, ^15^. Medical device companies, biomedical engineers and non‐governmental organizations should ensure that equipment that fits the context in LMICs is available commercially^1^, ^16^. The need for development of electrosurgery in LMICs has been highlighted^13^, ^17^, as well as laparoscopic equipment in conjunction with mobile technology, in order to decrease costs and reduce the number of devices required for laparoscopic surgery^18^, ^19^, ^20^.

The aim of this study was to explore the current availability, procurement, training, usage, maintenance and complications encountered during use of two frequently used types of equipment, the electrosurgical unit (ESU) and laparoscopic equipment, in hospitals in Africa.

## Methods

Attendees of the annual meeting of the College of Surgeons of East, Central and Southern Africa (COSECSA) in Maputo, Mozambique, in December 2017, and the annual meeting of the Surgical Society of Kenya (SSK) in Mombasa, Kenya, in March 2018, were asked to participate in a survey. Data on availability, procurement, training, usage, maintenance and complications related to the use of ESUs and laparoscopic equipment were collected. Respondents were asked to respond only to questions relating to the main hospital they worked at.

Visits to hospitals in Kenya were conducted to survey biomedical equipment technicians (BMETs) between February and April 2018. Data on training and maintenance of the ESU and laparoscopic equipment were collected.

Informed consent was obtained from all respondents. No personal details of respondents were recorded, so approval by an institutional review board was not required. All data were processed anonymously and archived to ensure privacy of the respondents.

All respondents were instructed that ‘laparoscopic equipment’ included the laparoscope, light source and insufflators. The ESU included the ESU generator and accessories (patient plate, monopolar electrode, bipolar handheld or foot pedal) that are required to use the ESU.

Maintenance records for ESU and laparoscopic equipment were examined to detail what type of maintenance was required, repairs needed and issues that BMETs were able to repair. Maintenance records reported the date, type of device, cause of failure and repair required between October 2015 and January 2018 in three hospitals in Kenya. Each original maintenance record was photocopied and stratified to type of surgical equipment, and analysed to indicate whether the BMETs were able to solve the maintenance issue.

## Results

**Figure 1:**
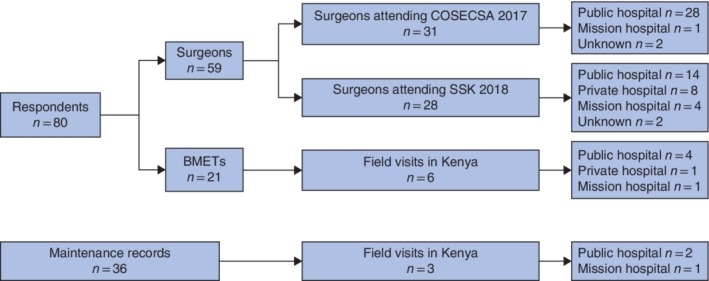
Overview of respondents and maintenance records COSECSA, College of Surgeons of East, Central and Southern Africa; SSK, Surgical Society of Kenya; BMETs, biomedical equipment technicians.

A total of 80 respondents were surveyed, of whom 59 were surgeons and 21 BMETs (*Fig*. 1). Thirty‐one surgeons who attended the COSECSA meeting represented hospitals in Burundi (1), Ethiopia (1), Kenya (6), Malawi (3), Mozambique (3), Namibia (3), Rwanda (3), Tanzania (3), Uganda (1), Zambia (4), Zimbabwe (1) and Swaziland (1); the country was unknown for one surgeon. Twenty‐eight surgeons who attended SSK 2018 represented hospitals in Kenya.

During the field visits conducted in Kenya, 21 BMETs were surveyed and 36 maintenance records on the ESU were collected.

### Use of the electrosurgical unit

An overview of the current availability, procurement, training, usage, maintenance and complications of ESUs according to the 59 surgeons is shown in *Table* 1. All surgeons had access to an ESU and 45 per cent had been trained in electrosurgery at medical school. Fourteen of the 59 surgeons (24 per cent) indicated that disposable accessories were cleaned in chemical solutions (such as glutaraldehyde) and reused.

**Table 1:** Availability, procurement, training, usage, maintenance and complications of the electrosurgical unit according to 59 surgeons

	*n*
**Availability**	
Access to an ESU	59 (100)
**Procurement**	
Purchased	27 (46)
Donated	8 (14)
Both purchased and donated	22 (37)
Unknown	2 (3)
**Training**	
Trained at medical school	26 of 58 (45)
Trained by medical device company	8 (14)
**Usage**	
Used ESU in bipolar and monopolar mode	39 of 57 (68)
Used ESU in monopolar mode only	15 of 57 (26)
Used ESU in bipolar mode only	3 of 57 (5)
Used coagulation	59 (100)
Used cut	57 (97)
Used fulgurate	7 (12)
Used blend	16 (27)
Reused disposable accessories	14 (24)
**Maintenance**	
Available	46 of 58 (79)
Performed by BMETs*	21 (36)
Outsourced (service contract, donation agency, partners abroad, etc.)*	5 (8)
**Complications**	
Encountered complications during use	29 (49)
+Burns	16 (27)
+Electrical shocks	7 (12)

Values in parentheses are percentages.

* These categories were not specified by all respondents who answered the question. ESU, electrosurgical unit; BMET, biomedical equipment technician.

*Table* 2 provides an overview of how electrosurgical equipment was procured in relation to reuse of accessories, access to in‐house maintenance, and complications. It shows that relatively more complications were encountered and more disposable accessories were reused when the ESU was donated rather than purchased. One surgeon who had purchased equipment indicated there was no access to in‐house maintenance, but that maintenance was provided by the medical device company.

**Table 2:** Overview of electrosurgical unit procurement in relation to reuse of disposable accessories, access to in‐house maintenance and complications

	Reuse of disposable accessories (*n* = 14)	No access to in‐house maintenance (*n* = 11)	Complications (*n* = 29)
Purchased (*n* = 27)	5	8	10
Donated (*n* = 8)	3	2	7
Both donated and purchased (*n* = 22)	6	1	11
Unknown (*n* = 2)	–	–	1

### Maintenance of the electrosurgical unit

All 21 BMET participants had access to an ESU and undertook maintenance. Almost half of the BMETs (10, 48 per cent) were trained at undergraduate level and six (29 per cent) were trained by the medical device company.

Of 36 maintenance records, 23 described repairs on the accessories (*Table* 3). All 23 issues with accessories were repaired successfully. Of 13 issues related to the ESU generator, nine were repaired successfully. In four situations, ESU generators were sent back to the manufacturer for further inspection. All 21 BMETs indicated that accessories (cables, connectors, patient plate and electrodes) were prone to breaking and required frequent maintenance. Over half of BMETs (12 of 21, 57 per cent) mentioned that power modules were prone to breakage.

**Table 3:** Maintenance records (*n* = 36) for the electrosurgical unit in three hospitals in Kenya

Part	Total	Repaired	Excerpt from record
Patient plate	14	14	Replacement of plate (*n* = 12)
			Soldered parts together (*n* = 2)
Monopolar electrode	6	6	Replacement (*n* = 1)
			Soldered new cables or other components (*n* = 5)
Foot pedal	3	3	Replacement of entire pedal (*n* = 1)
			Replacement of a faulty part (*n* = 2)
Generator	13	9*	Replaced display board (*n* = 1)
			Fitted new input filter (*n* = 3)
			Replaced faulty part by taking spare from an old machine (*n* = 1)
			Other repair (*n* = 4)

* Unsolved repairs were outsourced to the medical device company.

### Use of laparoscopic equipment

*Table* 4 presents an overview of the current availability, procurement, training, usage, maintenance and complications of laparoscopic equipment. Almost half of the surgeons who participated in the study (29 of 59, 49 per cent) had access to laparoscopic equipment. Complications during use were indicated by 52 per cent of the respondents (13 of 25), and included: fogging of the camera head, air leaks, inappropriate focus and instrument pairing, poor resolution, and conversion to open surgery because of limited experience. Nearly one‐third of surgeons (9 of 28, 32 per cent) indicated that they had difficulty obtaining carbon dioxide.

**Table 4:** Availability, procurement, training, usage, maintenance and complications of laparoscopic equipment

	*n*
**Availability**	
Access to laparoscopic equipment	29 of 59 (49)
**Procurement**	*n* = 29
Purchased	11 (38)
Donated	8 (28)
Leased	1 (3)
Both purchased and donated	7 (24)
Unknown	2 (7)
**Training**	
Trained at medical school	13 (45)
Trained by medical device company	13 of 28 (46)
**Maintenance**	
Available	16 of 27 (59)
Performed by BMETs*	5 (17)
Performed by service contracts*	5 (17)
**Complications**	
Encountered complications during use	13 of 25 (52)
Encountered difficulty obtaining carbon dioxide	9 of 28 (32)

Values in parentheses are percentages.

* These categories were not specified by all respondents who answered the question. BMET, biomedical equipment technician.

*Table* 5 provides an overview of how laparoscopic equipment was procured in relation to access to in‐house maintenance, complications and difficulty obtaining carbon dioxide. Participants using donated laparoscopic equipment had less access to in‐house maintenance and more difficulties obtaining carbon dioxide than those with procured laparoscopic equipment.

**Table 5:** Overview of procurement of laparoscopic equipment in relation to access to in‐house maintenance, complications and difficulties obtaining carbon dioxide

	No access to in‐house maintenance (*n* = 11)	Complications (*n* = 16)	Difficulty obtaining carbon dioxide (*n* = 10)
Purchased (*n* = 11)	3	6	2
Donated (*n* = 8)	4	5	3
Both donated and purchased (*n* = 7)	3	3	4
Leased (*n* = 1)	1	1	1
Unknown (*n* = 2)	–	2	–

### Maintenance of laparoscopic equipment

Most BMETs (16 of 21, 76 per cent) had access to laparoscopic equipment, and ten (48 per cent) indicated that they were able to maintain laparoscopic equipment. Eight BMETs (38 per cent) were trained by the medical device company on maintenance of laparoscopic equipment. Of the 16 BMETs who had access to laparoscopic equipment, most indicated that the light source (9, 56 per cent) and camera (8, 50 per cent) were most prone to breaking, followed by the insufflator (4, 25 per cent), ESU accessories (4, 25 per cent) and carbon dioxide seal (1, 6 per cent).

It was not possible to analyse maintenance records of laparoscopic equipment, mainly owing to unavailability of records.

## Discussion

This study revealed that, despite the availability of equipment (better for ESUs than for laparoscopic equipment), access to maintenance, training and consumables was not always in place. For example, ESUs were available to all surgeons and BMETs who participated in this study, but complications during electrosurgery, such as burns and shocks, were indicated by 27 and 12 per cent of surgeons respectively. Burns can occur because of improper attachment of the patient plate^21^, which can easily happen when disposable patient plate stickers are reused. Electric shocks can occur due to insulation failures, which can be caused by the chemicals used during the cleaning process.

Reuse of consumables in various LMIC settings has been reported previously^1^, ^17^, ^22^, and this practice was confirmed in the present study. Most maintenance records reported repairs on ESU accessories, with parts being soldered back together to enable reuse for as long as possible.

Almost 80 per cent of the responding surgeons had access to maintenance, and all of the BMETs carried out maintenance on the ESU. The analysed records revealed that maintenance often demanded small repair work on ESU accessories, but also included larger issues, such as the replacement of a display board. These repairs were all done successfully by the BMETs. This study showed that the repairs done by BMETs were a valuable asset in keeping the ESUs available in clinical practice, by solving small repair issues. Major repairs on the ESU generator were generally outsourced to the medical device supplier, donation agency, or other partners abroad. It is unknown whether it was lack of skills, tools or design of the ESUs that prevented in‐house maintenance.

Laparoscopic equipment was available to almost half of the surgeons and three‐quarters of the BMETs in this study. The findings highlighted complications with its use, notably difficulties with supplies of carbon dioxide. Gasless laparoscopic procedures have been proposed as an alternative to using carbon dioxide insufflation^23^. BMETs indicated that both the camera and the light source were prone to failure.

Procurement, training, use, maintenance and complications all require careful consideration when increasing availability of surgical equipment. This study has shown that not all of these aspects were covered consistently. This meant that equipment was available, but the service was difficult to sustain, for instance when new consumables were required or a repair was needed.

Approximately half of the available equipment in this study was purchased. A significant number of hospitals still relied on donations, reflecting relatively high purchasing costs, particularly of laparoscopic equipment in LMICs^3^. Difficulties with donations have been described before, when costs of consumables or maintenance have not been considered during the donation process^24^. Strategies for maintenance and purchase of accessories are as important as the donated equipment itself. Supply‐chain difficulties with ESU accessories, leading to reuse, and unreliable supplies of carbon dioxide (required only during laparoscopic surgery) were also important factors that limited the availability of a service. The use of non‐medical gases from local soft drink manufacturers^25^ or the implementation of gasless laparoscopy^23^ may be ways to overcome this last problem.

Of the surgeons who had laparoscopic equipment, 41 per cent did not have access to maintenance. Strategic investment in BMET training during implementation of new technologies should increase the availability of the surgical service^14^, ^15^.

Implementation of these new technologies and the associated equipment in LMICs presents different challenges from those in high‐income settings. Irregular power supplies, dust, high temperatures and medical device companies being outside the country are all well described problems^1^, ^2^, ^14^, ^17^, ^26^, ^27^.

This study has limitations. The majority of surgeons and all BMETs worked in Kenya, a country with more financial resources than many of the other countries taking part in the study. Surgeons who participated attended a scientific conference; this might have led to underrepresentation of rural hospitals that did not have the financial resources to let their employees attend these events. It would therefore seem likely that the availability of ESUs and laparoscopic equipment would be poorer elsewhere than in the hospitals in this study. The BMETs represented large urban hospitals in Kenya. The available skills and tools for maintenance might not be representative of more rural facilities in Kenya and hospitals in other LMICs. Only 36 maintenance records for a time span of 3 years were collected on the ESU in three large hospitals, and this might also have resulted in an underestimate of the number of maintenance issues associated with these devices. Digital maintenance reporting systems could increase access to maintenance data.

The wider adoption of laparoscopy in LMICs, along with associated technology such as an ESU, requires investment beyond initial equipment purchase and staff training in their use. This study has highlighted the need for an efficient supply chain for consumables and greater attention to maintenance issues to ensure delivery of a consistent and safe service for patients.

## Acknowledgements

This research was funded by the Delft Global Initiative of Delft University of Technology (grant number P70357).

*Disclosure*: The authors declare no conflict of interest.
